# Hyperuricaemia is associated with dyslipidemia but not HbA1c among type 2 diabetes mellitus patients in Botswana

**DOI:** 10.4102/ajlm.v8i1.786

**Published:** 2019-11-07

**Authors:** Ellen Gobusamang, Naledi G. Nyepetsi, Modisa S. Motswaledi, Ishmael Kasvosve

**Affiliations:** 1 Department of Medical Laboratory Sciences, Faculty of Health Sciences, University of Botswana, Gaborone, Botswana

**Keywords:** hyperuricaemia, diabetes mellitus, glycated haemoglobin, lipids, Africans

## Abstract

Medical records and residual samples from 334 type 2 diabetes mellitus patients attending a clinic in Gaborone, Botswana, during the period September–December 2016 were analysed for the effects of hyperuricaemia on biochemical markers of adverse outcomes. The patients were stratified as having hyperuricaemia (> 400 *µ*mol/L) or normal serum uric acid (≤ 400 *µ*mol/L). We compared glycated haemoglobin, triglycerides, low-density lipoprotein-cholesterol, high-density lipoprotein-cholesterol, total cholesterol and serum creatinine between the two serum uric acid categories. Hyperuricaemia was detected in 28% of patients (95% confidence interval 23.1–32.9) and was associated with increased serum triglycerides, triglyceride to high-density lipoprotein-cholesterol ratio and creatinine concentration, but not with glycated haemoglobin.

## Introduction

Epidemiologic studies suggest that hyperuricaemia plays a role in the aetiology and pathogenesis of a number of diseases including diabetes mellitus (DM).^1,2^ Serum uric acid (SUA) has a putative role in the development of cardiovascular disease.^3,4^ In a recent study conducted in South Africa, hyperuricaemia was associated with an increased risk of mortality among acute myocardial infarction patients.^5^ However, it remains unclear whether increased SUA concentration contributes to the aetiology or if it is a consequence of these conditions.

The role of SUA in type 2 diabetes mellitus (T2DM) is inconclusive. However, hyperuricaemia is associated with obesity and insulin resistance,^6^ and cross-sectional data have shown that hyperuricaemia is prevalent among T2DM patients.^7^ Other studies have also reported that hyperuricaemia increases the risk of T2DM.^8,9,10^ In a meta-analysis of 11 cohort studies, participants with hyperuricaemia had a 17% increased risk of developing diabetes per 1 mg/dL increase in SUA concentration.^9^ In another meta-analysis of eight prospective cohort studies, a 1 mg/dL increase in SUA resulted in a 6% increase in the risk of incident T2DM.^8^ Other studies have reported that hyperuricaemia predisposes individuals to the development of DM complications.^11^

Among DM patients, poor glycaemic control increases the risk of microvascular complications.^12^ The measurement of glycated haemoglobin (HbA_1c_) and lipids are used to monitor DM patients, and adverse changes are associated with the risk of DM complications. HbA_1c_ is a biochemical indicator of chronic glycaemia, and high values indicate poor control.^13^ The determination of total cholesterol, low-density lipoprotein (LDL) and high-density lipoprotein (HDL) cholesterol, and triglycerides provide valuable information for the prediction of coronary heart disease. Triglyceride concentration has an inverse relationship with HDL cholesterol concentration in DM patients.^13^ Both hypertriglyceridemia and low serum HDL cholesterol concentration independently increase the risk of developing coronary heart disease.^13^

Findings on the role of SUA in the pathogenesis of DM are inconsistent, and studies on associations between hyperuricaemia and the development of microvascular diseases among DM patients in African populations are lacking. In this study we determined the prevalence of hyperuricaemia and its association with HbA_1c_ and lipids in T2DM patients in Botswana.

## Methods

### Ethical considerations

The study was approved by the University of Botswana’s institutional review board, the Ministry of Health and Wellness Health Research Unit (permit number HPDME: 13/18/1 Vol X [619]) and the Research and Ethics Committee of Princess Marina Hospital (permit number PMH 5/79 [268-2-2016]). No individual consent was obtained from the patients.

### Study population

We conducted a cross-sectional study of residual samples from 334 T2DM patients attending a DM clinic in Gaborone City, during the period September–December 2016. The criteria for inclusion were age ≥ 18 years and a known history of T2DM. Minors (< 18 years) with T2DM were excluded from the study. The samples enrolled in the study were collected as part of clinical care and were de-identified prior to inclusion in the study, as recommended in the International Organization for Standardization’s 15189 standard. The clients’ identities were not revealed to study staff and confidential information was only made accessible to authorised persons.

### Laboratory investigations

Laboratory tests were performed on AU480 Chemistry Analyzer (Beckman Coulter Inc, Brea, California, United States). In some cases, the sample volume was insufficient to perform all the tests or the data were missing from the patient’s records. The analyser was calibrated and samples processed according to the manufacturer’s instructions using reagents provided by the manufacturer. Serum uric acid was measured using the uricase/peroxidase enzymatic method, and hyperuricaemia was defined as SUA > 400 *µ*mol/L. This cut-off is the upper reference limit used to define hyperuricaemia in Botswana. HbA_1c_ was measured using a latex agglutination assay. Briefly, a synthetic polymer containing multiple copies of the immunoreactive portion of HbA_1c_ agglutinates with latex coated with HbA_1c_-specific mouse monoclonal antibodies. Total cholesterol, HDL cholesterol, LDL cholesterol and triglycerides were determined by routine enzymatic methods. Creatinine was measured using a kinetic modification of the Jaffe procedure.^14^

### Statistical analysis

The results were analysed using the Statistical Package for Social Sciences version 24 statistical software (IBM Corporation, New York City, New York, United States). All quantitative variables were expressed as mean ± standard deviation or median with interquartile range (IQR). Categorical variables were expressed as proportions with estimated 95% confidence intervals (CI). Comparisons of HbA_1c_ concentration according to sex or SUA categories were conducted using an independent samples *t*-test, or the Mann-Whitney test was used to compare medians for non-parametric data. Results were considered statistically significant if *p* < 0.05. Box-and-whisker plots were used to show differences in serum creatinine concentration and HbA_1c_ according to uric acid category.

## Results

**FIGURE 1:**
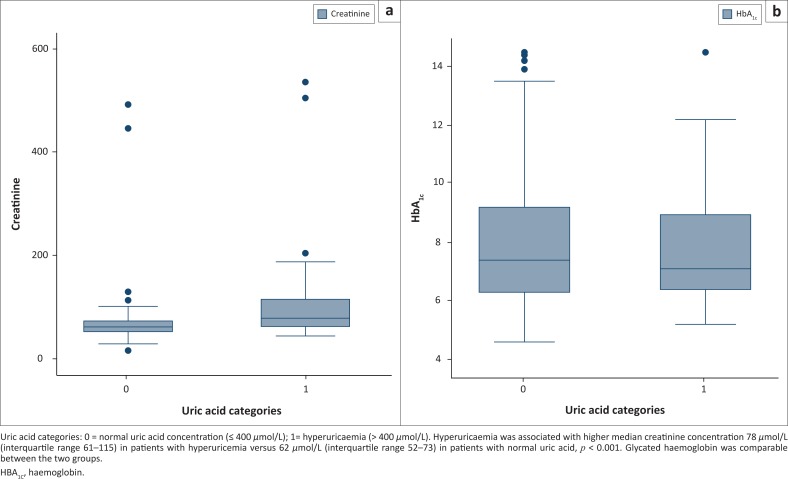
Comparison of (a) serum creatinine concentration and (b) glycated haemoglobin (HbA_1c_) according to uric acid category in type 2 diabetes mellitus patients receiving treatment in Gaborone, Botswana, September–December 2016.

Samples from 334 patients (236 women) with T2DM of unknown duration were studied. Ninety-two patients (28%, 95% CI 23.1–32.9) had hyperuricaemia and the prevalence was comparable between sexes (25% [95% CI 19.4–30.6] in women compared to 33% [95% CI 23.5–42.5] in men, *p* = 0.182). HbA_1c_ levels were comparable between SUA categories; median 7.1% (IQR 6.4–8.9) in T2DM patients with hyperuricaemia compared to median 7.4% (IQR 6.3–9.3) in T2DM patients with normal SUA, *p* = 0.379 (Table 1). Similarly, hyperuricaemia had no effect on total and LDL cholesterol levels, *p* ≥ 0.321. There was a tendency for lower HDL cholesterol among patients with hyperuricaemia, *p* = 0.090. Median triglyceride levels were higher among patients with hyperuricaemia (1.99 mmol/L [IQR 1.48–2.57] vs 1.72 mmol/L [IQR 1.19–2.57], *p* = 0.032); similarly, T2DM patients with hyperuricaemia had higher ratios of triglycerides to HDL cholesterol (median: 1.89 [IQR 1.33–2.79]) compared to T2DM patients with normal SUA concentration (median: 1.71 [IQR 1.00–2.44], *p* = 0.021). Hyperuricaemia was associated with higher median creatinine concentration, 78 *µ*mol/L (IQR 61–115) vs 62 *µ*mol/L (IQR 52–73) in patients with normal uric acid, *p* < 0.001 (Figure 1).

**TABLE 1:** Comparison of laboratory characteristics according to serum uric acid categories in type 2 diabetes mellitus patients receiving treatment in Gaborone, Botswana, September–December 2016.

Parameter	Normal uric acid *n* = 242	Hyperuricaemia *n* = 92	*p*
Age, years	-	-	0.235
Mean ± standard deviation	56 ± 14	58 ± 11	
Glycated haemoglobin	7.4†	7.1¶	0.379
%	6.3–9.3	6.4–8.9	
Creatinine concentration	62	78	< 0.001
*µ*mol/L	(52–73)	(61–115)	
Triglyceride concentration	1.72‡	1.99††	0.032
mmol/L	1.19–2.52	1.48–2.57	
LDL cholesterol, mmol/L	2.58 ± 0.87‡	2.70 ± 0.96††	0.321
HDL cholesterol	1.04§	0.99‡‡	0.090
mmol/L	0.90–1.28	0.86–1.19	
Total cholesterol, mmol/L	4.57 ± 1.22‡	4.72 ± 1.37††	0.371
Triglyceride/HDL cholesterol	1.71§	1.89‡‡	0.021
Ratio	(1.00–2.44)	(1.33–2.79)	
Uric acid concentration, *µ*mol/L	298 ± 65	481 ± 79	< 0.001

Note: In some cases, sample volume was insufficient to perform all the tests or the data were missing from the patient’s records.

Normal uric acid was defined as ≤ 400 *µ*mol/L and hyperuricaemia as serum uric acid > 400 *µ*mol/L. Values are expressed as mean ± standard deviation or median with interquartile range.

LDL, low-density lipoprotein; HDL, high-density lipoprotein.

† , *n* = 234.

‡ , *n* = 226.

§ , *n* = 206.

¶ , *n* = 90.

†† , *n* = 82.

‡‡ , *n* = 74.

## Discussion

The prevalence of hyperuricaemia among T2DM patients in Botswana is high and is comparable to findings in North Africa.^15^ In our study, hyperuricaemia was not associated with poor glycaemic control as measured by HbA_1c_. This is in contrast to data from the National Health and Nutrition Examination Survey III conducted nationwide in the United States.^16^ Our data corroborates findings from another study conducted on a cohort of newly diagnosed T2DM patients in China that reported no relationship between SUA and HbA_1c_ after adjusting for insulin concentration.^17^

Lipids are a risk factor for cardiovascular disease.^13^ In our study, T2DM patients with hyperuricaemia had significantly higher triglyceride concentrations and triglyceride to HDL cholesterol ratios. It has been suggested that a high triglyceride to HDL cholesterol ratio is a risk factor for coronary heart disease.^18^ The triglyceride to HDL cholesterol ratio can be used to predict the presence and degree of coronary atherosclerosis.^19^ Although high triglyceride to HDL cholesterol ratio has been reported to be a marker of insulin resistance,^20^ a study of African Americans did not corroborate this finding.^21^ Other lipids were not associated with hyperuricaemia in our study.

Hyperuricaemia was associated with an increased creatinine concentration. Although hyperuricaemia may simply be a marker of renal disease, there are studies which suggest that elevated SUA levels might contribute to the development and progression of renal dysfunction.^3^ Recently (2014), Toda et al. reported that hyperuricaemia was an independent risk factor for the development of chronic kidney disease,^22^ and in studies in Japan, elevated baseline SUA and increases in SUA increased the risk of developing chronic kidney disease.^23^ In another study, hyperuricaemia was associated with incident diabetic retinopathy among male patients with T2DM.^24^

### Limitations

We acknowledge that missing data on DM duration is a limitation to the interpretation of our findings, as the evolution of nephropathy and microvascular diseases may be influenced by the duration of DM. Adjusting for the duration of T2DM and the severity of DM might have sharpened our findings. In a study by Korpachev et al. in 2009, hyperuricaemia with decreased uric acid excretion was characteristic of severe DM, with a reduced kidney filtration rate.^25^ Conditions that reduce erythrocyte survival time may affect HbA_1c_ values. Although we did not screen for the effect of haemoglobinopathies or malaria on HbA_1c_, it is unlikely that these conditions were confounders in our study. Malaria transmission in southern Botswana is low to absent, and there is no literature on haemoglobinopathy in Botswana.

### Conclusion

In conclusion, hyperuricaemia was prevalent among T2DM patients in our study and was associated with elevated triglyceride concentrations, a high triglyceride to HDL cholesterol ratio and elevated serum creatinine concentration, but it was not associated with HbA_1c_ concentration. As recommended elsewhere, the monitoring of T2DM should include the measurement of SUA and the institution of interventions that lower SUA.^3^
